# Polypeptide-GalNAc-Transferase-13 Shows Prognostic Impact in Breast Cancer

**DOI:** 10.3390/cancers13225616

**Published:** 2021-11-10

**Authors:** Eugenia Fernandez, Luis Ubillos, Nabila Elgul, María Florencia Festari, Daniel Mazal, Otto Pritsch, Isabel Alonso, Eduardo Osinaga, Nora Berois

**Affiliations:** 1 Laboratorio de Glicobiología e Inmunología Tumoral, Institut Pasteur de Montevideo, Montevideo 11400, Uruguay; efernandez@pasteur.edu.uy (E.F.); eosinaga@pasteur.edu.uy (E.O.); 2 Servicio de Oncología Clínica, Hospital de Clínicas, Facultad de Medicina, Universidad de la República, Montevideo 11600, Uruguay; lubillos@fmed.edu.uy; 3 CASMU, Centro Asistencial del Sindicato Médico del Uruguay, Montevideo 11600, Uruguay; nelgul@pasteur.edu.uy (N.E.); or casecdtec@casmu.com (I.A.); 4 Departamento de Inmunobiología, Facultad de Medicina, Montevideo 11800, Uruguay; mfestari@fmed.edu.uy (M.F.F.); opritsch@fmed.edu.uy (O.P.); 5 Centro Hospitalario Pereira Rossell, Departamento de Anatomía Patológica y Citología del Hospital de la Mujer, Montevideo 11600, Uruguay; danielmazal@asse.com.uy; 6 Laboratorio de Inmunovirología, Institut Pasteur de Montevideo, Montevideo 11400, Uruguay

**Keywords:** breast cancer, GalNAc-T13, monoclonal antibody, immunohistochemistry, prognosis, *O*-glycosylation

## Abstract

**Simple Summary:**

The knowledge of the molecular mechanisms underlying breast tumorigenesis has allowed the identification of an increasing number of biomarkers, which have been correlated with cancer prognosis or used as predictors for specific treatment responses, thus improving the ability to individualize therapy. Protein *O*-glycosylation is dysregulated in breast cancer cells. Abnormal *O*-glycans have functional importance in cell adhesion, invasion, and metastasis. Polypeptide *N*-acetylgalactosaminyltransferases (GalNAc-Ts) family enzymes regulate the initial steps of mucin type *O*-glycosylation and may be responsible for the altered glycosylation observed in cancer. Previous reports have related GalNAc-T13 expression to cancer aggressiveness. In the present work, we produced a specific monoclonal antibody against GalNAc-T13 which is capable of recognizing this enzyme on formalin-fixed tissues. We observed a significant higher expression of this enzyme in metastatic samples compared with the corresponding primary tumors. Significantly, a high GalNAc-T13 score was associated with worse patient survival rates, thus supporting its prognostic potential.

**Abstract:**

Breast cancer is a public health concern and is currently the fifth cause of mortality worldwide. Identification of different biological subtypes is essential for clinical management; therefore, the role of pathologists is essential and useful tools for immunohistochemistry diagnosis are needed. Polypeptide-GalNAc-transferases are emerging novel biomarkers related to cancer behavior and GalNAc-T13, correlated with aggressiveness in some tumors, is an interesting candidate. Few monoclonal antibodies reacting with native proteins, and not affected by fixation and paraffin embedding, have been reported. The aim of this work was to develop a useful monoclonal antibody anti-GalNAc-T13 and to assess its potential significance in breast cancer diagnosis. We evaluated 6 human breast cancer cell lines, 338 primary breast tumors and 48 metastatic lymph nodes and looked for clinical significance correlating GalNAc-T13 expression with patients’ clinical features and survival. We found high GalNAc-T13 expression in 43.8% of the cases and observed a significant higher expression in metastatic lymph nodes, correlating with worse overall survival. We hypothesized several possible molecular mechanisms and their implications. We conclude that GalNAc-T13 may be a novel biomarker in breast cancer, useful for routine pathological diagnosis. Elucidation of molecular mechanisms related to aggressiveness should contribute to understand the role of GalNAc-T13 in breast cancer biology.

## 1. Introduction

Breast cancer reached the highest global cancer incidence in 2020, with an estimated 2.3 million new cases per year, representing 11.7% of all cancer cases, and 685,000 deaths; it is currently the fifth cause of cancer-related deaths worldwide [1]. Metastatic disease is the main cause of death in these patients, and resistance to treatment is the focus of oncological treatments. In the era of personalized medicine, knowledge of tumor biology allows the development of prognostic and predictive biomarkers, thus helping in clinical decisions [2]. Nevertheless, despite progress in the diagnosis of circulating tumor cells and in the molecular classification of the aggressive forms, many molecular diagnostic methods still have substantial technological requirements, resulting in only a few of them being currently available in most laboratories.

The role of pathologists is essential, since immunohistochemical biomarkers are used to classify breast cancer into different biological subtypes and to guide treatment [3]. The identification and validation of biomarkers is critical before general clinical use. Immunohistochemical assessment of several antigens in recent years has demonstrated the practical utility of avoiding the use of more sophisticated and expensive molecular techniques. A major concern in developing breast cancer biomarkers is tumor heterogeneity, a dynamic process driven both by intrinsic effects of the tumor cells, as well as extrinsic effects from the surrounding microenvironment, contributing together to malignant tumor progression, metastasis development and therapy resistance [4]. Thereby, novel molecular diagnostic tools are still required, and multiple molecular validation studies comprising immunohistochemistry of different promising breast cancer biomarkers are needed for the correct management of patients.

Glycans displayed at the cellular surface are crucial for the phenotype of cancer cells, regulating cell–cell and cell–matrix interconnections, modulating cell signaling and environmental interactions [5]. Differences in glycosylation patterns between normal cells and tumor cells are allowing the identification of novel cancer biomarkers, development of anti-cancer drugs and the enhancement of tumors responses to immunotherapy [6,7]. A long history of glycan binding studies with plant lectins and antibodies has led to the development of commonly clinically used biomarkers that recognize glycan or glycopeptide structures, such as CA15-3, CA125, CA19-9, and B72.3 [8]. CA15-3 recognizes an aberrant O-glycosylated epitope on MUC-1, frequently overexpressed in breast cancer. Protein core exposure increases adhesion of tumor cells and is associated with higher tumor grades and worse prognosis [9]. 

The polypeptide-N-acetylgalactosaminyltransferases family (GTs), composed by 20 members in humans, catalyzes the first step of mucin-type O-glycosylation. These enzymes have differential expression patterns in normal tissues and show different expression profiles in normal vs. tumor cells [10]. It is proposed that the expression of these enzymes could affect the glycosylation of specific proteins at the cellular surface (e.g., EGFR, IGFR-1, MUC1), which could contribute to the neoplastic phenotype. While GalNAc-T1 and GalNAc-T2 are constitutively expressed in breast tissues, other GalNAc-Ts, that are aberrantly expressed in breast cancer, have been proposed as tumor markers. Analyzing glycan-related genes expression in breast cancer subtypes, GALNT3 and GALNT6 were found to be the genes that varied significantly between the five subtypes. In addition, GALNT6 has been found to be overexpressed in ductal carcinoma in situ (DCIS) [11]. These results confirm our previous findings showing GalNAc-T6 expression by immunohistochemistry for the first time in most DCIS and early tumors, suggesting an early role in breast carcinogenesis [12], which was later confirmed by other researchers, who also suggested a role in tumor escape from immune recognition [13,14]. Glycosylation by GalNAc-T6 is essential to stabilize MUC1, thus inducing an anti-adhesive effect which could facilitate metastasis development via beta-catenin/MUC1-C signaling pathway [15,16]. In the same way, smoke-induced MUC1-N glycosylation by GalNAc-T6 correlated with carcinogenesis in lung cancer [17], and GalNAc-T6 expression assessed by immunohistochemistry was significantly associated with advanced TNM stage, and independently predicted worse overall survival in lung adenocarcinomas [18]. In this regard, while searching for disseminated tumor cell biomarkers in breast cancer, we previously found that GALNT6 expression assessment in bone marrow aspirates was helpful to identify a subset of patients with worse survival rates among the negative lymph node group, suggesting its role as prognostic marker [19]. GalNAc-T14 is another isoenzyme associated with aggressiveness in breast, ovarian, lung cancer and neuroblastoma [20]. This isoenzyme was previously demonstrated to be over-expressed in breast cancer by immunohistochemistry [21], and its expression was also specifically associated with breast cancer relapse to the lung [22].

Among the other GTs isoenzymes, GALNT13 has been correlated with cancer aggressiveness. This isoform was initially characterized as highly restricted to the nervous system [23] and is considered to be a close paralog of the ubiquitous GALNT1 isoenzyme [24]. Although the 20 isoenzymes catalyze the same reaction, subtle differences can be displayed between them. GalNAc-T13 is able to synthesize clusters of tri-Tn [23]. We previously found it is overexpressed in a human metastatic neuroblastoma model [25]. In addition, evaluating bone marrow involvement and comparing cytology with GALNT13 expression and three other developing markers, we found that this isoenzyme showed the best correlation with poor survival. In the same way, Matsumoto et al. reported high metastatic potential of a murine Lewis lung cancer model related to GalNAc-T13 glycosylation [26], and increased expression of this enzyme was correlated with worse prognostic in lung cancer patients [27]. Considering these results, we hypothesize that GalNAc-T13 could also be a biomarker for breast cancer and our aim in the present work is to evaluate its expression by immunohistochemistry and to correlate it with clinical-pathologic features. We found that high GalNAc-T13 expression was associated with lymph node metastasis and a more aggressive behavior.

## 2. Materials and Methods

### 2.1. Anti-GalNAc-T13 Monoclonal Antibody (MAb) Production

A specific synthetic peptide derived from GalNAc-T13 (RSLLPALRAVISRNQE, accession number AJ505991; purchased from Biosynthesis Inc., Lewisville, TX, USA) was selected in the region displaying high variability between this isoenzyme and other GalNAc-Ts family members. Four subcutaneous inoculations with 100 µg of this peptide conjugated to keyhole-limpet hemocyanin were performed at 2-week intervals for BALB/c mice immunization. Isolated spleen cells were fused with mouse myeloma cells SP2/O, and supernatant screening and antibody titer were performed by ELISA, using microtiter plates coated with the same peptide sequence conjugated to bovine serum albumin, as previously described [12]. Several obtained positive clones underwent further characterization.

### 2.2. Analysis of MAbs Specificity by Surface Plasmon Resonance

Interactions between the anti-GalNAc-T13 antibodies and small synthetic peptides (overlapping the whole sequence chosen for immunization) were analyzed by surface plasmon resonance experiments on a BIAcore 3000 instrument (GE Healthcare, Danderyd, Sweden). Purified MAbs were coupled to an activated carboxymethylated dextran CM-5 sensor surface (SA sensor chip, GE Healthcare, Danderyd, Sweden). The peptides were diluted in HBS-EP buffer (10 mM HEPES, 150 mM NaCl, 3 mM EDTA, 0.005% Surfactant P20, pH 7.4) and were passed over the sensor-chips. All experiments were run in duplicate at a 30 μL/min flow rate, a contact time of 180 s and a dissociation time of 360 s, with the biosensor instrument thermostat at 25 °C. After dissociation, the sensor chip was regenerated by injecting 10 mM glycine–HCl (pH 2.5) at the end of each experiment. All data processing was carried out using the BIAevaluation 4.1 software provided by BIAcore. We choose MAb T13.5 for further analysis.

### 2.3. Western Blot

Recombinant human GalNAc-T1 and GalNAc-T13, produced and purified as previously described [28], were tested by Western blotting using MAb T13.5. Briefly, similar quantities of both enzymes were run on a NuPage Novex BisTris 4–12% gel (Thermo Fisher Scientific, South San Francisco, CA 94080, USA) and blotted onto a nitrocellulose membrane (Amersham; Aylesbury, UK) for 50 min, followed by blocking of residual protein-binding sites by incubation in 5% BSA in Tris-buffered saline (TBS). After washing with TBS-0.05% Tween 20, the membrane was incubated with the primary antibody (T13.5 culture supernatant) overnight at 4 °C. After three washes with TBS-0.05% Tween 20, the membrane was incubated with goat anti-mouse alkaline phosphatase-conjugated (Sigma-Aldrich, St. Louis, MO, USA) for 1 h at room temperature, and then revealed with 5-bromo-4-chloro-3-indolyl phosphate/nitro blue tetrazolium substrate. 

### 2.4. Clinical Samples and Immunohistochemistry Staining

Breast cancer patients who underwent surgical resection of their primary tumor at CASMU (Non-profit Professional Medical Private Institution, Montevideo, Uruguay), and signed informed consent, were included in this study for GalNAc-T13 retrospective expression evaluation by immunohistochemistry (*n* = 22). Tissue microarrays (TMA) were purchased from US Biomax, Inc. (Rockville MD 20849, USA) (multiple organs tumor: MC5003b; breast cancer: BR10010f, BR1202a, BC081120f, HBre-Duc150Sur-01, *n* = 370). Detailed clinical and pathological information, including pathological tumor, node, and metastasis stage, receptor status and follow-up information of overall survival (OS) rates, were retrieved from the electronic clinical records and information provided by TMA supplier.

For immunohistochemical staining, 5 μm thick histology sections were de-paraffinized, hydrated, analyzed for GalNAc-T13 expression using T13.5 culture supernatant as the primary antibody and mouse specific HRP/DAB (ABC) detection IHC kit (abcam, Cambridge, UK) following the provided protocol. Briefly, endogenous peroxide was blocked with provided reagent for 15 min, followed by washes and the provided protein blocking solution incubation for 15 min, to abolish nonspecific background staining. The primary antibody was incubated overnight at 4 °C, and, after four washes, incubated at room temperature for 10 min with biotinylated goat anti-mouse IgG. After several washes, streptavidin peroxidase was incubated 10 min at room temperature, followed by additional washes and incubation for 5 min with DAB chromogen solution freshly prepared. Slides were then counterstained with hematoxylin, washed, dehydrated, and mounted. As a negative control, we replaced the primary antibody with phosphate-buffered saline. Immunohistochemical expression was quantified using a final score obtained by multiplying a 4-value intensity score (0–3 for negative, weak, moderate, and strong, respectively), and the percentage of positive tumor cells. A composite score formed by the product of the marking intensity and its extension was developed, ranging from a minimum of 0 to a maximum of 300. Two observers (D.M. and N.B.), blindly and independently, evaluated all slides. The cases were reviewed to reach a consensus if there were discrepancies found in the evaluation.

### 2.5. Cell Lines

Cell lines were purchased from American Type Culture Collection (ATCC): MCF-7 (RRID: CVCL_0031), MDA-MB-231 (RRID: CVCL_0062), MDA-MB-157 (RRID: CVCL_0618), T47D (RRID: CVCL_0553), SK-BR-3 (RRID: CVCL_0033), BT-474 (RRID: CVCL_0179), A549 (RRID: CVCL_0023), and HeLa (RRID: CVCL_0030). A549 T13-/- was generated in our laboratory using Crispr/Cas9 technology in collaboration with Henrik Clausen (Copenhagen Center for Glycomics, University of Copenhagen, Denmark; unpublished results). All cell lines were in vitro cultured in vitro at 37 °C in DMEM supplemented with 10% fetal bovine serum, 1% glutamine and 1% pyruvate, at 5% CO_2_ humidified atmosphere.

### 2.6. Reverse Transcription-Polymerase Chain Reaction (RT-PCR and qRT-PCR)

Total RNA was extracted from cell lines with Tri-Reagent (Sigma-Aldrich, St. Louis, MO, USA) according to the manufacturer’s instructions and stocked at −80 °C until use. One µg of RNA was reverse transcribed using by M-MLV reverse transcriptase (Invitrogen™, ThermoFisher Scientific). The reaction mixture consisted of 200 U of enzyme, 2 µL of 10 mol/L of each deoxynucleotide triphosphate (dNTPs) and 200 ng of random hexamers (Invitrogen™, Thermo Fisher Scientific, South San Francisco, CA 94080, USA), in a 20 µL total reaction volume. After 1 h of incubation at 37 °C the mixture was heated to 85 °C, snap-cooled and stored at −20 °C. A fragment of 600 bp of the β2M (β2-microglobulin) gene was amplified to verify cDNA quality, using the following specific primers: B2MF, 5′-ATGTCTCGCTCCGTGGCCTTAG-3′; B2MR: 5′-AAGTTGCCAGCCCTCCTAGAGC-3′. The reaction conditions consisted of the addition of 1 µL of cDNA to a final 25 µL PCR reaction volume, containing 1× provided enzyme buffer, 2 mM MgCl_2_, 200 µM dNTPs, 300 nM of each primer and 1 unit of Taq DNA polymerase recombinant (Invitrogen™, ThermoFisher Scientific, South San Francisco, CA 94080, USA). In this case, 35 cycles were performed as follows: 1 min at 95 °C, 1 min at 62 °C and 1 min at 72 °C, followed by an extension step of 5 min at 72 °C. Amplification of *GALNT13* sequence (accession number AJ505991) was performed by nested PCR as follow:-First round amplifies a 425 bp fragment in a final 25 µL PCR reaction volume containing 1× provided enzyme buffer, 3 mM MgCl_2_, 200 µM dNTPs, 300 nM of each primer (GALNT13-F, 5′-ACATCTATCCGGACTCCC-3′; T13-Rev, 5′-TCATGTGCCCAAGGTCATGTTCC-3′) and 1 unit of Taq DNA polymerase recombinant (Invitrogen™, ThermoFisher Scientific, South San Francisco, CA 94080, USA). The amplification conditions were 30 cycles of 30 s at 94 °C, 30 s at 60 °C and 1 min at 72 °C, followed by an extension step of 5 min at 72 °C. One µL of first round product was subsequently used to perform a second round of 20 cycles in the same amplification conditions, obtaining a 183 bp fragment with the following specific primers: T13-10F, 5′-AAATCCGAACCGATGACTTG-3′; T13-11R, 5′-TAGGCACCATTTTGTCTTCTT-3′. The PCR mixture was the same as for the first round, although the MgCl_2_ final concentration was 2 mM. In this case, 20 µL of PCR products were analyzed by electrophoresis on 2% agarose gels by direct visualization after ethidium bromide staining. The quantification of *GALNT13* expression in cell lines was performed by RealTime PCR (cobas 4800, Roche), using the following specific primers: GALNT13-F, 5′-ACATCTATCCGGACTCCC-3′; GALNT13-R, 5′-GGCCCATGTTGTCTAAA-3′. One microliter of a 1 in 10 dilution of cDNA was added to a final 20 µL PCR reaction volume containing 10 µL SyBrGreen reagent, 0.4 µL ROX high reagent and 0.15 µL of 20 nM of each primer. The amplification conditions were 35 cycles of 15 s at 94 °C, 20 s at 56 °C and 3 s at 72 °C. Melting curve was performed to analyze the obtained PCR products.

### 2.7. Immunocytochemistry and Immunofluorescence

Cells plated on glass coverslips were washed with PBS, fixed in cold methanol-acetone 50% *v*/*v* for 10 min and stored a −20 °C until use. Coverslips were then defrosted and rehydrated in PBS. GalNAc-T13 expression was assessed using T13.5 culture supernatant as the primary antibody and mouse specific HRP/DAB (ABC) detection IHC kit (abcam, Cambridge, UK) following provided protocol (see Section 2.4). For negative control, we replaced the primary antibody with phosphate-buffered saline. For immunofluorescence we followed the same procedure for cell lines preparation and primary antibody incubation, followed by secondary antibody Alexa Fluor^®^ 488 goat-anti mouse IgG (A11029–Invitrogen) incubation for 1 h. at room temperature and after three washes, monolayers were counterstained with DAPI 1 µg/mL, mounted in PBS-glycerol 50% and analyzed by epifluorescence microscopy.

### 2.8. Statistics

The relationship between the expression of GalNAc-T13 and the clinical-pathological variables of the patients and the included normal breast controls was evaluated using the Fisher’s exact test. A significant association was considered when *p*-values were <0.05. Univariate survival analysis was performed using the Kaplan–Meier method and compared with the log-rank test. The relationship between GalNAc-T13 expression and survival was determined with the nonparametric Mantel–Cox log-rank test to compare the survival distribution. All statistical tests were two-tailed. The proportional hazard assumption was tested by Schoenfeld’s method and plotting (-log(-log S(t))). All statistical calculations were performed using GraphPad Prism Sofware v9.00 (GraphPad Software, Inc.).

## 3. Results and Discussion

### 3.1. Production of a MAb Specific for GalNAc-T13

**Figure 1:**
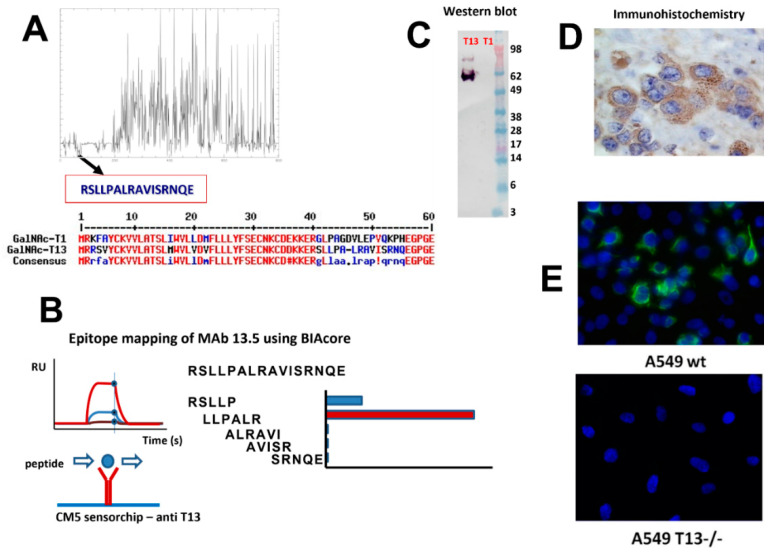
Anti-GalNAc-T13 monoclonal antibody production. (**A**) Selection of a differential sequence specific of GALNT13, avoiding cross reaction with GALNT1: Multiple sequence alignment calculated with MUSCLE v3.8.31 [32], plot performed using ‘plotcon’ from the EMBOSS suite [33], using a window size of 1. Selection of 16 aa sequence (residues 40–56) displaying no similarities with GalNAc-T1 was confirmed by MultAlin [34]. (**B**) Epitope mapping of MAb T13.5 by BIAcore using small synthetic peptides overlapping the whole sequence used for immunization. We deduced that the epitope matches with the LLPAL sequence. (**C**) Western blot performed with recombinant GalNAc-T13 and GalNAc-T1. (**D**) Confirmation of GalNAc-T13 expression by immunohistochemistry in formalin-fixed, paraffin embedded neuroblastoma tissues (magnification 400×). (**E**) Immunofluorescence of A549 wt and A549 T13-/- cell lines (magnification 200×).

With the aim of obtaining a specific tool to evaluate GalNAc-T13 expression by immunohistochemistry, we sought to develop a MAb. Given the great homology among GalNAc-Ts isoenzymes, we selected a GalNAc-T13 specific sequence in the region displaying the highest variability, specially avoiding similar sequences to GalNAc-T1, which is the isoenzyme most closely related to GalNAc-T13. This is a challenging task, considering that both isoenzymes have 84% sequence homology [24]. Only two MAbs anti-GalNAc-T13 have been reported, and both display cross reaction with GalNAc-T1 [29], so a specific MAb recognizing GalNAc-T13 in formalin-fixed paraffin embedded (FFPE) tissues could be a favorable contribution to pathological diagnosis. We previously produced an anti-GalNAc-T6 MAb with an appropriate performance in such conditions, and we assessed its expression in breast cancer [12], as well as gastric and colon cancer [30,31]. Following the same working strategy, we first looked for isoenzyme’s homology using EMBOSS open software for sequence alignment and we selected a specific sequence (Figure 1A). Splenocytes from immunized mice with the selected synthetic peptide were fused to a murine myeloma cell line, which gave rise to several clones. After mapping epitopes by BIAcore with overlapping peptides, we selected MAb T13.5 for further characterization (Figure 1B). GalNAc-T13 specificity was confirmed by Western blot with recombinant GalNAc-T1 and -T13 (Figure 1C), and by immunohistochemistry with FFPE tissues from neuroblastoma, where GalNAc-T13 is overexpressed [24]. Immunostaining exhibits a perinuclear granular pattern, typical of Golgi apparatus staining (Figure 1D). We also compared GalNAc-T13 expression in the lung cancer A549 cell line, which expresses GalNAc-T13, and A549 T13-/- cell line generated by Crispr/Cas9 technology (unpublished results). We confirmed abolishment of GalNAc-T13 expression in the A549 T13-/- cell line (Figure 1E).

To our knowledge, the anti-GalNAc-T13 MAb generated in this work together with our previously developed anti-GalNAc-T6 MAb [12], are the only reported MAbs reacting with native proteins, and not affected by fixation and paraffin embedding. It is probable that the short peptide sequence used as an immunogen, located at the stem region of the enzymes, favored the linear recognition, while other immunization strategies, with longer peptides or recombinant proteins, could favor conformational epitope recognition which can be affected by histological processing. 

### 3.2. GalNAc-T13 Is Expressed in Breast Cancer

**Figure 2:**
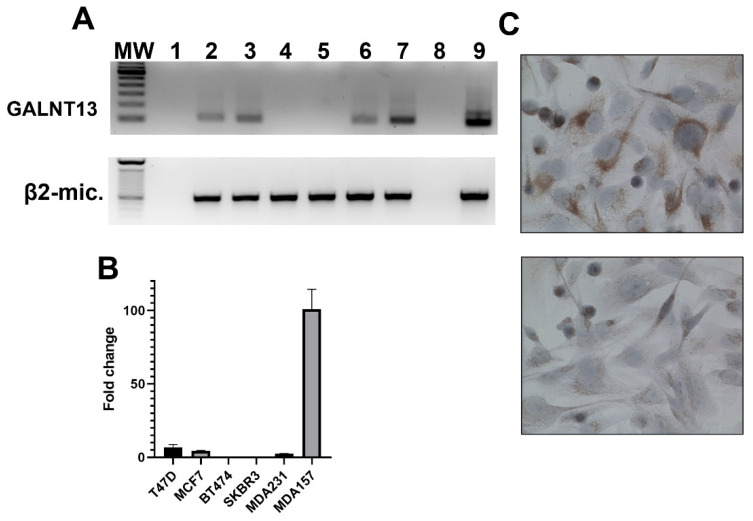
GalNAc-T13 expression in human breast cancer cell lines. (**A**) Nested reverse-transcription PCR amplifying a 105 bp fragment of GALNT13 mRNA and one-round reverse-transcription PCR amplifying a 596 bp fragment of β2-microglobulin as cDNA quality control: MW, 100 bp DNA ladder (Thermo Fischer); 1, master-mix negative control; 2, T47D; 3, MCF7; 4, BT474; 5, SKBR3; 6, MDA-MB-231; 7, MDA-MB-157; 8, ultrapure water as negative control; 9, Hela cell line as positive control. (**B**) Quantitative real-time PCR for the same breast cancer cell lines. (**C**) Immunocytochemistry evaluating GalNAc-T13 expression at the protein level in MDA-MB-157 cell line in the upper image and negative control in the lower image (magnification: 400×).

Tissue microarrays MC5003b (US Biomax, Inc. Rockville, MD 20849, USA), containing samples of 20 different types of tumors (and their matching normal control tissues), were screened for GalNAc-T13 expression. We found differential expression of the isoenzyme in breast cancer in comparison with normal breast tissue. With the purpose of characterizing GalNAc-T13 expression in cell lines exhibiting different features, we evaluated mRNA expression by nested RT-PCR and real-time-RT-PCR in representative cell lines of different molecular subtypes of breast cancer (T47D and MCF-7, luminal A; BT474, luminal B; SK-BR-3, HER2; MDA-MB-231 and MDA-MB-157, triple negative breast cancer) [35]. We found GALNT13 expression by PCR in luminal A (T47D, MCF-7) and both triple negative cell lines (MDA-MB-231, MDA-MB-157) (Figure 2A nested RT-PCR; 2B RealTime-RT-PCR). Expression at the protein level was confirmed in the most expressive cell line (MDA-MB-157) by immunocytochemistry (Figure 2C).

**Figure 3:**
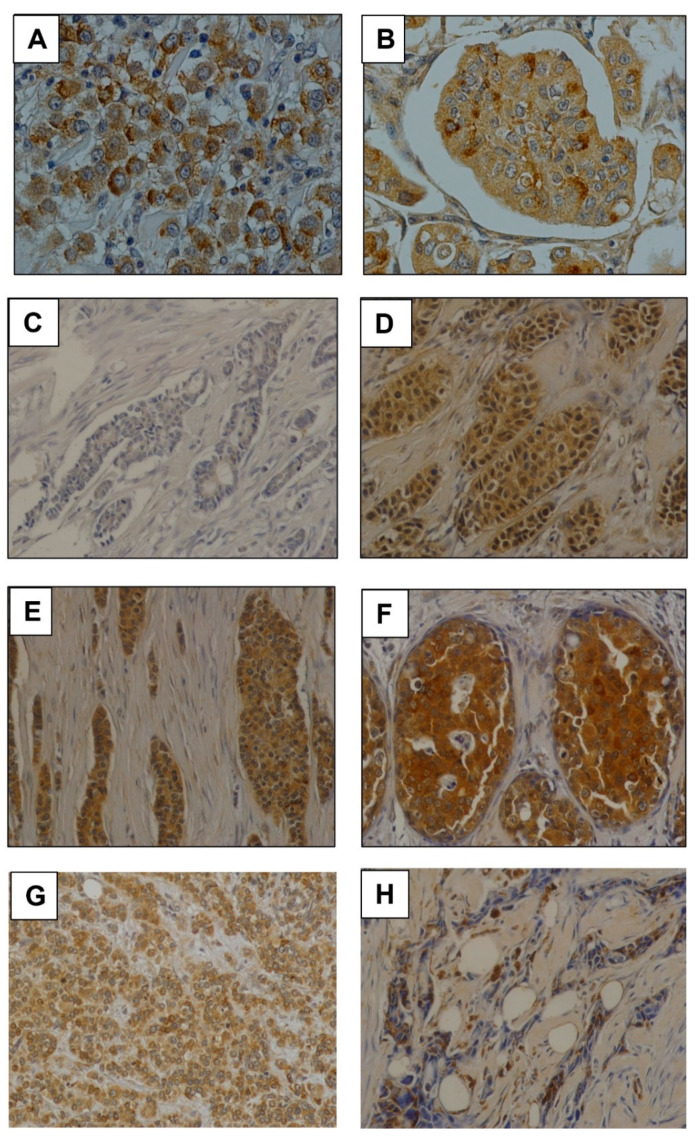
Monoclonal antibody T13.5 immunostaining in breast cancer. (**A**,**B**) Immunostaining of anti-GalNAc-T13 MAb showing diffuse and granular cytoplasmic pattern and several perinuclear reinforcements. Both pictures have been taken at 400× magnification. (**C**–**F**) Examples of immunostaining intensity: (−), (+), (++) and (+++), respectively. (**G**) Immunostaining in a high percentage of cells. (**H**) Focal immunostaining in a low percentage of cells. Pictures (**C**–**H**) have been taken at 200× magnification.

Moreover, GalNAc-T13 expression was evaluated in breast cancer tumors from CASMU patients and TMA (US Biomax), *n* = 338. Most tumors were invasive ductal carcinomas, but seven lobular carcinomas, seven medullar carcinomas and two breast tumors with neuroendocrine features were also analyzed. We mainly observed a diffuse cytoplasmic staining, sometimes exhibiting a dot pattern or perinuclear reinforcement (Figure 3A,B), concordant with Golgi apparatus location. Score for GalNAc-T13 expression was obtained on the basis of staining intensity (Figure 3C,F), multiplied by the percentage of tumor-stained cells (Figure 3G,H), and the median score was established as the cut-off value for the low/high GalNAc-T13 expression allocation of the samples.

**Figure 4:**
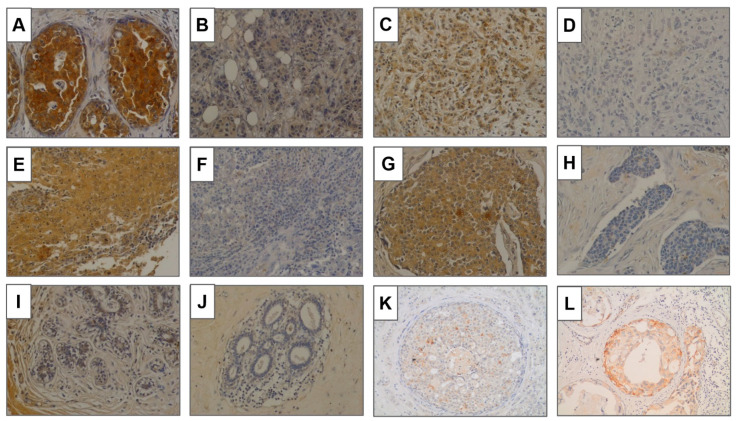
GalNAc-T13 expression in breast tissues. (**A**,**B**) Positive and negative expression, respectively, in invasive ductal carcinoma. (**C**,**D**) Positive and negative expression in lobular carcinoma. (**E**,**F**) Positive and negative expression in invasive carcinoma with medullary features. (**G**,**H**) Positive and negative expression in carcinoma with neuroendocrine features. (**I**,**J**) Normal breast tissue adjacent to breast cancer. (**K**,**L**) Ductal carcinoma in situ of low grade and high grade. Magnification of all pictures: 200×.

GalNAc-T13 expression was observed in different histological types, but not in most normal breast samples (Figure 4A–H). We analyzed 13 normal breast tissues adjacent to breast cancer and only two showed a weak positivity. Over all TMAs we observed five ductal carcinoma in situ (DCIS), four of low grade and one of high grade. The low-grade lesions exhibited a faint to moderate GalNAc-T13 expression in more than 90% of cells. The immunostaining of the high-grade DCIS was more intense, similar to that of the surrounding invasive tumor (Figure 4). 

Table 1 shows the results of the immunohistochemical study related to the clinical features of the patients (*n* = 338).

High GalNAc-T13 expression was found in 148/338 cases (43.8%). Most tumors were invasive ductal carcinomas and 44.7% of these (144/322) showed high expression of the enzyme. Among other histological types, we analyzed seven lobular carcinomas, two of them expressing GalNAc-T13, as well as one of seven medullary carcinomas and one of two tumors exhibiting neuroendocrine features. No statistical significance was found for GalNAc-T13 high expression compared with histological grade, tumor size and hormonal receptors. However, the enzyme is highly expressed in Her2 (+) tumors, with a significant difference compared to Her2 (−) tumors (*p* = 0.002). In the same way, when comparing GalNAc-T13 expression among different breast cancer molecular types, we also found statistical significance for high expression in the Her2 subtype (*p* = 0.022). 

### 3.3. GalNAc-T13 Is an Aggressiveness Marker in Breast Cancer

**Figure 5:**
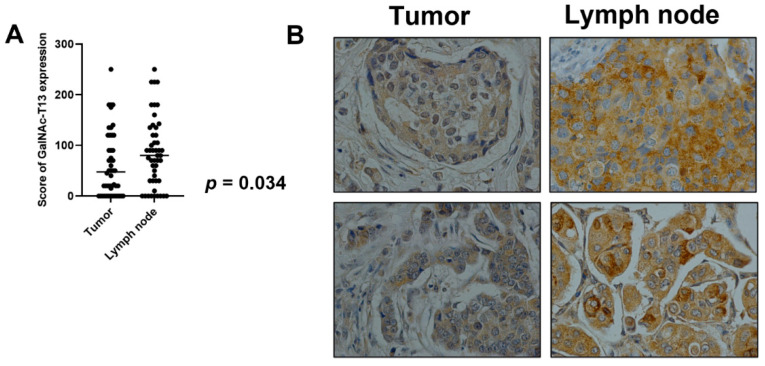
GalNAc-T13 expression in primary breast tumors and lymph node metastasis. (**A**) Comparison of mean expression of GalNAc-T13 by primary tumors and metastatic lymph nodes (*p* = 0.034). (**B**) GalNAc-T13 immunostaining in primary tumors and corresponding lymph node metastasis (400× magnification).

In 48 cases, we could compare GalNAc-T13 immunostaining in the primary tumor with that in the corresponding metastatic lymph nodes. We observed a significantly higher expression of GalNAc-T13 in metastatic samples compared with the corresponding primary tumors (*p* = 0.034) (Figure 5A). Only 30% of primary tumors exhibiting score 0 in GalNAc-T13 expression also showed a score of 0 in metastatic lymph nodes. Some examples of immunostaining in paired samples are shown in Figure 5B. 

**Figure 6:**
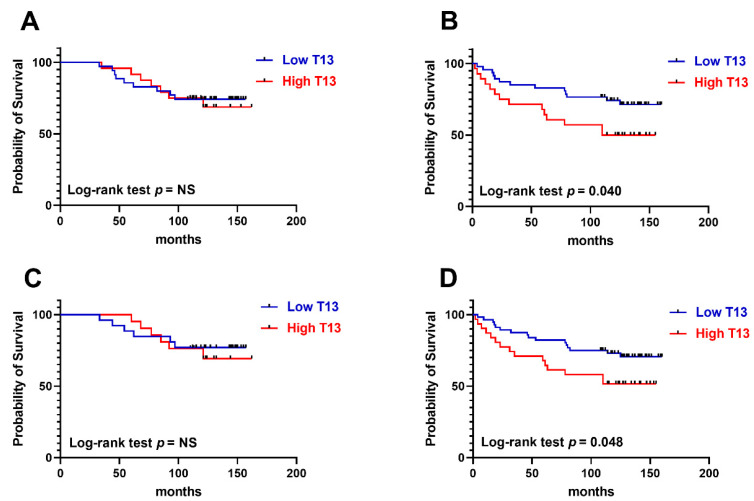
Kaplan–Meier overall survival rates related to GalNAc-T13 expression. (**A**) Early stage tumors (stages I and IIA) (*n* = 59). (**B**) Advanced stage tumors (stages IIB and III) (*n* = 75). (**C**) Patients without lymph node involvement (*n* = 47). (**D**) Patients with metastatic lymph nodes (*n* = 87).

Overall survival was recorded for 134 cases and Kaplan–Meier curves were established comparing GalNAc-T13 high and low expression in different clinical feature situations. GalNAc-T13 high expression was significantly correlated with less overall survival in lymph-node-positive patients and high stage tumors (Figure 6).

In our work, we identified a relationship between the expression of GalNAc-T13 and the expression of HER2, as well as with the HER 2 subtype. In turn, the high expression of GalNAc-T13 showed a clear pejorative effect on survival, both, in the stages with greater locoregional compromise (stage IIb and III), as well as in those patients with axillary involvement. Although these scenarios must usually be treated with complementary chemotherapy, there is still a range of variability in which to look for prognostic factors (as is the case), potentially predictive of response to complementary treatments. This is already established in less advanced settings, where genetic tests such as Oncotype, Mammaprint or Prosigna, are employed to predict the usefulness of treatments with possible side effects such as chemotherapy. An important perspective at the clinical level is to identify the previously mentioned molecular markers and their relationship with the tumor level, as well as their predictive value (not only prognostic). It is important to demonstrate that GalNAc-T13 expression or its relationship with other markers would imply a more or less aggressive treatment.

Two recent works demonstrated significant differences in gene expression of primary breast cancer tumors compared with lymph node metastases, concluding that understanding these genomic changes may provide useful knowledge of the metastatic process and also an opportunity for novel biomarker identification [36,37]. This fact agrees with our results demonstrating significantly higher GalNAc-T13 expression in metastatic lymph nodes than in primary tumors. Indeed, high GalNAc-T13 expression among patients with involved lymph nodes correlated with worse overall survival. The molecular mechanisms explaining this relationship between GalNAc-T13 status and the aggressiveness of the disease or its worse prognosis remain to be elucidated. Published studies focused on the modulation of cell adhesion functions by GalNAc-Ts, as well as on their influence in the degradation of connective tissue, which could be linked to the higher expression of the enzyme at the lymph node metastases. Various GalNAc-Ts were shown to affect leukocyte adhesion through modulation of E and P selectin [38,39]; GalNAc-T3 was found to modulate the activities of metalloproteinases [40]. GalNAc-T6 was also found to regulate molecular E-cadherin and β-catenin cell adhesion in breast cancer [15]. Therefore, it is reasonable to hypothesize that GalNAc-T13 may alter the ability to invade and metastasize by affecting cell–cell adhesion and cell–stroma interactions in breast cancer and this may be evidenced by the increased expression at the lymph node metastases and by its pejorative prognosis. In this way, Matsumoto et al. demonstrated that trimeric Tn antigen produced by GalNAc-T13 induces high metastatic potential in a murine lung cancer model [26], and deepening on the molecular mechanisms, they demonstrated that GalNAc-T13 induced trimeric-Tn on syndecan-1, which forms a complex with integrin α5β1 and MMP-9, enhancing invasion and metastasis [41]. Syndecan-1, also known as CD138, is a transmembrane proteoglycan expressed in normal and malignant tissues. In breast cancer, syndecan-1 expression was proposed as a prognostic marker in a compartment dependent manner, with cytoplasmic positivity being linked to aggressive cancer and stromal expression being linked to a more favorable prognosis [42]. Furthermore, Sayyad et al. demonstrated that syndecan-1 expression is associated with brain metastases, correlating with lower disease-free survival, especially in TNBC [43]. The authors reported that syndecan-1 supports breast cancer cells transmigration through the blood brain barrier by cytokines action, concluding that the elucidation of this mechanism will allow the development of novel therapeutic strategies. 

We previously analyzed the in vitro glycosylation of a large panel of 180 synthetic peptides by using GalNAc-T13 [28]. Among them, several were peptides belonging to glycoproteins related to biological behavior in cancer. Interestingly, seven of these proteins have been reported as being expressed in breast cancer and could be related to molecular subtypes or tumor biology (annexin A1, APO E, endoplasmin, factor V, frizzled 6, carboxypeptidase D and neuroregulin 3). ANXA1 (annexin A1) expression in breast cancer was found in a large cohort of patients from the international Breast Cancer Association Consortium, mainly associated with patients exhibiting well-known bad prognosis features (poorly differentiated tumors, triple negative, young age, BRCA1/2 mutations) [44]. Indeed, Silva-Oliveira et al. also suggested the interest of ANXA1 expression as prognostic marker in Her2+ patients, helping in the stratification of patients for the treatment choice [45]. Moreover, ANXA1 expression in the microenvironment of TNBC may promote Treg-cell mediated immune suppression, leading to breast tumor growth, resulting in an interesting target for further investigation of breast cancer immunotherapy [46]. Cumulative evidence has linked apolipoproteins (APOs) in diverse mechanisms and many vital functions in cancer and suggested their potential utility in diagnosis biomarker development and targeted therapy [47]. In breast cancer, different APOs exhibit dissimilar behavior depending on the location (serum or tumor). In this regard, circulating APO E has been positively associated with aggressiveness while APO E expression in tumors showed negative correlation with breast cancer development [48]. Interestingly, Flowers et al. reported differences in APO E glycosylation in a location dependent manner, with plasma isoforms being less glycosylated than cerebrospinal fluid isoforms, and differences in C-terminal glycosylation could have functional consequences in lipoprotein binding [49]. Growing evidence highlighting the influence of changes in APO E glycosylation on breast cancer, among other gynecological diseases, underlines the importance of understanding this process, including the role of a variety of enzymes, providing opportunities for diagnostic and therapeutic strategies [50]. In this context, we can hypothesize that GalNAc-T13 could play a role in such process, thus deepening in the underlying molecular mechanisms is required. Another peptide glycosylated by GalNAc-T13 is derived from endoplasmin, also known as GRP94 and HSP90b1. This protein is an endoplasmic reticulum resident chaperon that participates in protein folding. Nevertheless, it can also be translocated to the cell surface and participates in the regulation of several functions, including cell growth, adhesion and immunity. High GRP94 expression level has been found to be an independent and unfavorable prognostic indicator of breast cancer survival [51] and in vitro experiments have demonstrated that GRP94 knockdown in breast cancer cells reduced invasive capacity and enhanced sensitivity to drugs [52]. Overexpression of GRP94 on the plasma membrane enhances dimerization and phosphorylation of HER2, promoting signaling and tumor growth [53], and growing evidence suggests that GRP94 at cell surface could be a potential antibody therapy target in breast cancer [54]. However, no glycosylation aberration has been linked to the functional effects of GRP94. Conversely, an exhaustive study of Factor V glycosylation demonstrated a great variability of O-glycans all over the molecule [55]; interestingly, the most diversified glycoforms were found on Thr805, included in the sequence glycosylated by GalNAc-T13 in our previous report. Overexpression of Factor V has been reported in breast cancer, especially in more aggressive subtypes, but it is related to improved overall survival [56]. Tumors with high expression of *F5* exhibit an increased inflammatory infiltration, with lymphoid and myeloid cells. This is probably related to a better immune anti-tumor response, which could explain the better prognostic of such tumors, suggesting a potential opportunity for novel therapeutic strategies [57]. Frizzled 6 belongs to a family of transmembrane receptors for Wnt signaling proteins, frequently amplified in breast cancer, mainly in triple-negative molecular type [58]. It has been related to chemoresistance in neuroblastoma cells as well as to lung cells proliferation [59]. These tumors are where we have previously found that GalNAc-T13 expression was related to aggressiveness [25]. Carboxypeptidase-D (CPD) is upregulated in breast cancer by hormonal influence and promotes cell survival by increasing nitric oxide production. Mac Donald et al. reported high CPD staining by immunohistochemistry in triple negative and Her2 positive breast tumors, and Kaplan–Meier plot analyses revealed that high CPD mRNA expression correlated with a poorer relapse-free survival rate in patients with triple negative tumors, suggesting this pathway as a potential therapeutic strategy [60]. Increasing evidence relates proteases glycosylation with functional activity [61]; thus, research in this field could provide novel opportunities in translational oncology. Finally, neuroregulin 3 (NRG3) is a soluble secreted ligand which binds and activates some members of the epidermal growth factor receptor family, expressed in normal and malignant breast epithelial cells [62], and is implicated in mammary gland development and carcinogenesis. Multiple splice variants have been described for neuregulin proteins, and NRG3 contains multiple sites for O-linked glycosylation but no sites for N-linked sugar addition [63]. Although NRG expression was not associated with survival of breast cancer patients, it has been suggested as a predictive biomarker for targeted therapies [64]. It is well known that abnormal O-glycosylation may be the outcome of an imbalance between glycosyltrasferases expression and substrate availability. Exposure of tumor associated antigens as a consequence of aberrant O-glycosylation has been largely associated with oncogenesis by several mechanisms affecting cell adhesion, invasion, and metastatic process. In this context, our hypothesis is that GalNAc-T13, aberrantly expressed in breast cancer, together with the overexpression of different proteins related to breast cancer biology as mentioned above, could play a potential role in mammary oncogenesis. Therefore, the dilucidation of the involved molecular mechanisms is imperative.

## 4. Conclusions

In the present work, we report the production of a mAb anti-GalNAc-T13 that is able to recognize this enzyme on formalin-fixed tissues, which may be useful in routine pathological studies. We found that GalNAc-T13 was highly expressed in metastatic lymph nodes compared with the respective primary tumors. This marker correlated with poor clinical outcomes in breast cancer patients, suggesting that this enzyme could be a novel biomarker of more aggressive subtypes of breast cancer and a potential candidate for targeted therapy. Further work is needed to elucidate the biological role of GalNAc-T13 in breast cancer, to analyze the molecular mechanisms regulating this gene expression, and to identify the acceptor substrates of this enzyme potentially involved in the metastatic behavior in this type of cancer.

## Figures and Tables

**Table 1:** Correlation of GalNAc-T13 expression with the clinical features of the patients.

	Patients	Low GalNAc-T13 ^1^	High GalNAc-T13	
	*n*	*n* (%)	*n* (%)	*p* ^2^
Total	338	190 (56.2)	148 (43.8)	
Mean age 51.3 (range: 27–83)				
Histological type				NS ^3^
Invasive ductal carcinoma	322	178 (55.3)	144 (44.7)	
Others	16	12 (75)	4 (25)	
Histological Grade				NS
1	17	12 (70.6)	5 (29.4)	
2	211	105 (49.8)	106 (50.2)	
3	51	31 (60.8)	20 (39.2)	
T				NS
T1	46	32 (69.6)	14 (30.4)	
T2	226	125 (55.3)	101 (44.7)	
T3	43	19 (44.2)	24 (55.8)	
T4	20	11 (55)	9 (45)	
N				NS
N0	153	86 (56.2)	67 (43.8)	
N+	182	101 (55.5)	81 (44.5)	
Hormonal Receptors				
ER ^4^ (-)	139	75 (54)	64 (46)	NS
(+)	189	112 (59.3)	77 (40.7)	
PR ^5^ (-)	182	97 (53.3)	85 (46.7)	NS
(+)	144	89 (61.8)	55 (38.2)	
Her2 (-)	230	142 (61.7)	88 (38.3)	0.002
(+)	87	37 (42.5)	50 (57.5)	
Molecular types				0.022
Luminal A	106	64 (60.4)	42 (39.6)	
Luminal B	92	55 (59.8)	37 (40.2)	
Her2	41	14 (34)	27 (66)	
TNBC ^6^	78	46 (59)	32 (41)	
Stage				NS
I	25	15 (60)	10 (40)	
II	209	121 (57.9)	88 (42.1)	
III	101	51 (50.5)	50 (49.5)17	

^1^ Cutoff established at median score (40). ^2^ Fisher’s exact test: *p* < 0.05 was considered significant. ^3^ Not significant. ^4^ Estrogen Receptor. ^5^ Progesterone Receptor. ^6^ Triple Negative Breast Cancer.

## Data Availability

The data presented in this study are available on request from the corresponding author.
